# Probiotics in Inflammatory Bowel Diseases and Associated Conditions

**DOI:** 10.3390/nu3020245

**Published:** 2011-02-17

**Authors:** David R. Mack

**Affiliations:** Children’s Hospital of Eastern Ontario, 401 Smyth Road, Ottawa, Ontario K1H 8L1, Canada; Email: dmack@cheo.on.ca; Tel.: +1-613-737-7600 (ext.) 2516; Fax: +1-613-738-4854

**Keywords:** Crohn’s disease, ulcerative colitis, pouchitis, spondyloartopathy, arthralgia, sclerosing cholangitis, maintenance, induction, remission

## Abstract

A complex set of interactions between the human genes encoding innate protective functions and immune defenses and the environment of the intestinal mucosa with its microbiota is currently considered key to the pathogenesis of the chronic inflammatory bowel diseases (IBD). Probiotics offer a method to potentially alter the intestinal microbiome exogenously or may provide an option to deliver microbial metabolic products to alter the chronicity of intestinal mucosal inflammation characterizing IBD. At present, there is little evidence for the benefit of currently used probiotic microbes in Crohn’s disease or associated conditions affecting extra-intestinal organs. However, clinical practice guidelines are now including a probiotic as an option for recurrent and relapsing antibiotic sensitive pouchitis and the use of probiotics in mild ulcerative colitis is provocative and suggests potential for benefit in select patients but concerns remain about proof from trials.

## 1. Introduction

A recent large-scale metagenomic sequencing study revealed the presence of a core set of bacterial species shared between individuals [1]. Individuals harbor at least 160 bacteria species, among which 75 and 57 are common to more than 50% and 90% of individuals, respectively. It is now evident that the gut microbiota is composed of 2 components: A core set of bacteria common to everyone and another set whose composition varies between individuals and this intestinal microbiota gene set is some 150 times larger than the host human gene complement. The existence of this variable set likely arises from functional redundancy in the bacterial world, but becomes pertinent when discussing administration of probiotic strains at high doses over long periods of time in patients with Inflammatory Bowel Diseases (IBD). 

IBD consists of chronic and relapsing inflammatory diseases of the intestines classically comprising of two similar yet distinct subtypes: ulcerative colitis (UC) and Crohn’s disease (CD). UC and CD differ by the intestinal localization and features of the inflammation. UC by definition is continuous inflammation starting in the rectum and restricted to the colon while CD inflammation can occur anywhere in the gastrointestinal tract, often with skip lesions. Microscopic lesions are restricted to the mucosa layer for UC while affecting the full thickness of the intestinal wall for CD [2].

Careful epidemiological studies have demonstrated that CD is increasing in some western countries and most worrisome is a steep rise in younger children [3]. Unfortunately, there is no medical cure for IBD and despite considerable research efforts the cause of IBD is uncertain. Recent genome wide association studies (GWAS) in both adults and pediatrics [4,5,6] have been highly successful in identify novel pathways in the pathogenesis of IBD and yet these GWAS have only identified 25% of the genetic risk for developing IBD and have yet to produce therapeutic results. 

Environmental factors also are linked with development of IBD such as amount of fibre and fats in diets [7,8], active and passive tobacco smoking [9,10] and vitamin D deficiency [11,12]. Indeed, certain living environments also seem to play a role as evidenced by studies linking the development of CD with living on a farm within the first 6–12 months of life [13,14] and second generation South Asian immigrants moving to Canada’s west coast were found to have a very high and an increasing incidence of IBD [15]. There was not access for all of the above studies to biologic specimens to assess how environmental factors might change host-microbe interaction, gut microbe balance or gene-environment interaction.

Some of the best evidence that the gut microbiota plays a key role in IBD comes from animal model studies. Although the experimental animal models of IBD do not exactly mimic human UC and poorly mimic CD, these studies have shown that the development of the disease is dependent on the presence of resident bacteria. A key finding from animal models is that in a number of separate animal models with induced, spontaneous or genetically engineered disease, chronic colonic inflammation is initiated and perpetuated in the presence of resident enteric bacteria, whereas germ-free (sterile) conditions prevent or dramatically attenuate the development of disease [16,17]. More recently, the loss of the transcriptional factor T-bet in mice, which regulates the differentiation and function of immune system cells, was shown to promote the bacterial community to become colitogenic [18]. Moreover, the induced colitis could be communicated to other genetically intact hosts by vertical transfer of the colitogenic microbiota [18]. This experiment clearly demonstrated that the composition of the microbial community could directly cause colitis (in the same genetic background). Moreover, in children both the number of immune responses and the magnitude of immune response to various microbial antigens involving antibodies to the *Escherichia coli* outer-membrane porin C (OmpC), *Saccharomyces cerevisiae* (ASCA) and anti-flagellin antibodies (Anti-CBir1) were predictive of aggressive CD phenotypes [19]. 

Thus, it is becoming clearer that the complex interactions between microbial, genetic, immune, and environmental factors are critical in the pathogenesis of IBD. The proposed mechanisms in the genetically susceptible host that lead toward aggressive cellular immune responses in response to components of the microbiota and the development of experimental colitis in mutant animals include loss of epithelial cell barrier function, overexpression of pro-inflammatory mediators in different effector T lymphocyte subsets (Th1 and Th17, Th2), deficient protective and regulatory signals and/or abnormal antigen presentation [16,17]. This leads to a dysregulated immune response directed against the intestinal microbiota and results in a disruption of the intestinal microbiota equilibrium. The etiology of IBD can therefore be conceptualized as an aberrant immune response to a component or components of the gut microbiota potentially triggered following an environmental insult in a genetically susceptible individual. It remains unknown whether the human gut microbiome triggers, is altered as a secondary response to the intestinal inflammation or maintains the chronicity of intestinal inflammation that is the hallmark of IBD. Nevertheless, as the majority of IBD susceptibility genes identified are involved in regulation of innate or adaptive immunity and maintenance of the intestinal mucosal barrier, it is apparent that the microbiome plays a critical role in the development and natural history of disease. The addition of probiotics to this complex microenvironment may thus affect at a number of levels including direct effects on the immunologic reaction of the host, indirectly lessen the immunologic reaction of the host by improving the mucosal barrier function to lessen interaction with the host immune system, displacement of deleterious microbes from luminal-mucosal interface or alter metabolic consequences of the microbiome.

The use of probiotics has been proposed for providing benefits to human health for a long time but in recent years there has been increased interest for their use in inflammatory bowel disease due to the microbiome role in IBD pathogenesis [20]. Probiotics are being ingested by patients with IBD sometimes through the advice of the physician but mostly self-prescribed as a form of alternative medicine [21,22]. The reasons for their usage seem to be mostly related to severity of disease, side effects of treatments and health beliefs [21,22,23]. Recent reports compared to even a few years ago would suggest increase in the use such that up to 50% of patients with IBD or parents of children with IBD are at least trying probiotics if not taking on a regular basis and parents giving them to their affected children [21,22,23,24,25,26,27]. The aim of this chapter is to review information that is available at the current time.

## 2. Ulcerative Colitis

### 2.1. Treatment of Active Inflammation in Ulcerative Colitis

A systematic review with data analysis has been performed on the first randomized trials (See Table 1, references [28,29,30,31]) involving probiotics for induction of remission for ulcerative colitis [32]. Due to the significant differences in probiotics, outcomes and trial methodology and as outlined in Table 1, a formal meta-analysis was not preformed. They are small trials with approximately 10–52 participants in treatment arms for participants with mild to moderate disease activity. The probiotics were given as single and blends of microorganisms, probiotic in combination with a prebiotic fructooligosaccharide/inulin mixture and combined with allopathic medicine. 

Following their systematic review, Mallon *et al.* [32] concluded that addition of a probiotic to conventional therapy did not improve overall remission rates in patients with mild to moderate ulcerative colitis but the addition of probiotics may reduce disease activity. The data were analyzed using intention to treat for the three studies that measured proportion of patients achieving remission [28,30,31]. 

For these three studies:

- probiotics (Yakult™) + 5-ASA had similar effectiveness to placebo + 5-ASA for induction of remission [28]: probiotic 40%, placebo 30%, OR 0.64 (95% CI 0.10 to 4.10);
- probiotics (VSL#3™) + balsalazide had similar effectiveness to placebo + balsalazide for induction of remission [30]: probiotic 80%, placebo 70%, OR 0.58 (95% CI 0.18 to 1.91).
- probiotics (*E. coli* Nissle 1917) + steroids had similar effectiveness to mesalazine + steroids for induction of remission [31]: probiotic 68.4%, mesalazine 74.6%, OR 1.35 (95% CI 0.6 to 3.04).

As comparison, in a trial of 268 UC patients with moderate severity 4.8 g of delayed release oral mesalamine was found to have clinical benefit in 70% and superior to a response rate of 59% for those using a lower dose of 2.4 g of a delayed release oral mesalamine for moderate UC disease activity [33].

**Table 1:** Randomized Trials of probiotics as therapy of active UC.

Participants (# Treated)	Trial Design	Probiotic (Strains)	Dosing (CFU/day)	Trial Length (Weeks)	References
20 (10)	EBRPC	Blend Probiotic (Yakult™)	1 × 10^10^	12	[28]
16 (8)	DBRPC	Single Probiotic + Prebioticmn (*B. longum*)	4 × 10^11^	4	[29]
90 (30)	R	Blend Probiotic (VSL#3™)	9 × 10^11^	8	[30]
102 (52)	DBRDD	Single strain (*E. coli* Nissle)	1 × 10^11^	12	[31]
29 (14)	DBRPC	Blend Probiotic (VSL#3™)	1 × 10^11^/kg	52	[34]
120 (80)	R	Probiotic ± Prebiotic (*B. longum*)	2 × 10^9^	4	[35]
90 (70)	DBRPC	Single strain (*E. coli* Nissle)	1–4 × 10^9^	2–8	[36]
147 (77)	DBRPC	Blend Probiotic (VSL#3™)	7.2 × 10^12^	12	[37]

EBRPC: Endoscopy blinded, randomized, placebo-controlled; DBRPC: Double-blind randomized placebo-controlled; R: Randomized; DBRDD: Double-blind, randomized, double-dummy; Blend: combination of two or more probiotic organisms; Yakult™: *B. breve*, *B. bifidum* and *L. acidophilus*; VSL#3™: *L. acidophilus*, *L. plantarum*, *L. paracasei*, *L. bulgaricus*, *B. breve*, *B. longum*, *B. infantis*, and *S. thermophilus*.

There have been some additional randomized placebo-controlled trials studies since the Cochrane systematic review (Table 1 [34,35,36,37]). In a trial in children with moderate-to-severe disease VSL#3™ or placebo was administered along with corticosteroids and mesalamine. The corticosteroid dose (1 mg/kg/day to a maximum of 40 mg/day) and mesalamine (50 mg/kg/day) dose were those commonly used. The corticosteroids were tapered after a month if subjects were in remission. In this study, remission was achieved in 13 of 14 participants (92.8%) treated with VSL#3™ and IBD therapy and in 4 of 15 patients (36.4%) treated with placebo and IBD therapy (*p* < 0.001). This result must be taken in context the response rate to corticosteroids and mesalamine in the placebo treated group. As a comparison, in a multicentre North American registry reporting the outcome of children with newly diagnosed UC, 60% of those treated with corticosteroids were in remission at 3 months [38].

Using a quality of life measure Fujimori and colleagues [35] studied patients on stable doses of aminosalicylates and/or prednisolone for at least 4 weeks in remission or had mildly active UC. They reported that that only those patients taking a combination of a prebiotic and *B. longum* had an improvement (*p* = 0.03) whereas those subjects on the individual components (either prebiotic alone or probiotic individually) did not. The probiotic was taken once daily and the prebiotic twice daily and the study was not double dummy controlled and thus one wonders about a lack of blinding effect on outcome. The only measure of disease activity was C-reactive protein on a small number from each group. 

The response to rectal enemas of *E. coli* Nissle in subjects with distal proctitis of moderate activity was studied by Matthes *et al.* [36]. The concentration of the probiotic was 10^8^ CFU/mL and subjects with mild to moderate disease activity were randomized to receive enemas once daily containing either 10 mL, 20 mL, 40 mL or placebo that was volume matched the three different enema volumes used in the *E. coli* Nissle groups. Permissible concomitant therapies included loperamide drops to improve retention capacity for enemas, and oral UC maintenance treatment with aminosalicylates or steroids at a constant level for at least two weeks prior to the study. A disease activity index was used to measure response and if there was no response at 2 weeks, they were classified non-responders but otherwise could continue up to 8 weeks of therapy. In contrast to per protocol analyses, intention to treat analysis revealed the number of responders was not significantly higher in the *E. coli* Nissle group than in the placebo group (*p* = 0.4430) in this Phase II study.

In a multicenter, randomized, double blind, placebo-controlled trial from India, Sood *et al.* [37] studied the blend probiotic VSL#3™ in adults with mild-to- moderate UC. Participants were assigned randomly to groups that were given 3.6 × 10^12^ CFU VSL#3 (*N* = 77) or placebo (*n* = 70) twice daily for 12 weeks. A primary end point of 50% decrease in the Ulcerative Colitis Disease Activity Index (UCDAI) at 6 weeks was achieved in a greater number of those receiving probiotic (32.5%) than the group given placebo (10%) (*p* = 0.001). A secondary end point of remission by 12 weeks was achieved in 33 subjects given probiotic (42.9%) compared with 11 subjects given placebo (15.7%, *p* = 0.001). 

### 2.2. Probiotics as Maintenance Therapy in Ulcerative Colitis

Only a few probiotic products either combined as blends (*n* = 2) or administered as single strain monotherapy (*n* = 4) have been studied in UC maintenance trials with 3 of the single probiotic trials utilizing *E. coli* strain Nissle 1917. With a background of up to 70% relapse rate over a 1-year period for those with ulcerative colitis not taking any form of maintenance therapy [39], many of the trials have been for one year (Table 2) and studied remission rates in comparison with 5-aminosalicylate products [31,40,41]. One of these 12-month probiotic *versus* 5-aminosalicylate trials was initiated with active UC patients [31] and followed those achieving remission for a 12-month period. In this study the relapse rates were high in both the group maintained with *E. coli* Nissle 1917 and those maintained on 1.5 g of daily mesalazine (67% and 72%, respectively). The other 12-month trials were initiated in participants with quiescent disease. In these studies, maintenance of remission rates varied between 45% and 75% [40,41,42] and studies in those receiving 5-aminosalicylates as a control group had a similar maintenance of remission rate as the probiotic intervention group [40,41]. Interestingly in the trial comparing monotherapy *L. rhamnosus* strain GG, monotherapy mesalamine (2.4 g per day) and combination probiotic and mesalamine, no synergistic benefit was derived form combination therapy but all three groups had equivalent rates for maintenance of remission [41]. The studies comparing probiotic with 5-aminosalicylates have used different total daily amounts (1.5–2.4 g per day). Nevertheless, currently there is not currently clinical evidence of a direct dose-dependent maintenance benefit above 1.6 g daily dosing of 5-aminosalicylate [43]. 

In the small trial in children [34], the blend probiotic VSL#3™ reported 3 of 14 (21.4%) patients treated with VSL#3™ and their other IBD therapy and 11 of 15 (73.3%) patients treated with placebo and IBD therapy relapsed within 1 year of follow-up (*P* = 0.014; *RR* = 0.32; *CI* = 0.025–0.773). It is not obvious from the trials done to date that there is any advantage to blends of probiotics as compared to single probiotics and there are no comparative trials to answer this question.

**Table 2:** Randomized Trials of probiotics used as maintenance therapy for ulcerative colitis.

Participants (# Treated)	Trial Design	Probiotic (Strains)	Dosing (CFU/day)	Trial Length (Months)	References
103 (50)	DBRDD	Single strain (E*. coli* Nissle)	5 × 10^10^	3	[44]
83 (39)	DBRDD	Single strain (*E. coli* Nissle)	1 × 10^11^	12	[31]
21 (11)	R	Blend (Yakult^®^)	1 × 10^10^	12	[42]
327 (162)	DBRDD	Single strain (*E. coli* Nissle)	5 × 10^9^	12	[40]
187 (127)	R	Single strain (*L. rhamnosus* GG)	1.8 × 10^10^	12	[41]
29 (14)	DBRPC	Blend Probiotic (VSL#3™)	1 × 10^11^/kg	12	[34]

Same footnotes as Table 1.

### 2.3. Pouchitis

Proctocolectomy with ileal pouch-anal anastomosis may be required in some UC patients because their disease was medically intractable or they developed secondary dysplasia or cancer. Pouchitis or inflammation of the ileal reservoir created during the procedure may develop in between 15 and 50% in patients. It is the most common complication of the surgery and although the exact etiology is not clear host genetic factors, local pouch issues and the microbiota contained within the pouch are thought to be involved [45,46].

Most patients will develop this problem in the first year and antibiotics can be an effective form of therapy in many [45,46] but for those that do not respond the term antibiotic-resistant is applied and these patients can be chronically active requiring other forms of therapy [46]. For some, antibiotics improve the pouchitis but there is a relapsing course of the pouchitis following the discontinuation of antibiotics. As antibiotics can provide relief for most with pouchitis, a basic assumption has been the importance of the microbiota of the pouch in the development and chronicity of pouchitis. Thus, alteration of the microbiota by addition of probiotics was considered. Subsequently, probiotics for treatment of acute pouchitis, prevention of initial onset of pouchitis and prevention of relapsing pouchitis have all been evaluated. 

#### 2.3.1. Probiotics as Treatment of Pouchitis

Trials for treating mild/moderate pouchitis are few with small numbers of adult participants. Kuisma *et al.* [47] recruited 20 patients (10 intervention arm) for a DBRPC trial of *L. rhamnosus* GG 2 × 10^10^ CFU/day for 3 months. Those patients with chronic, active pouchitis were excluded. The Pouchitis Disease Activity Index [48] was utilized for evaluation of clinical effect. Prior to study entry, the mean PDAI was in the mild range (8.0 ± 0.8) and no there was no difference following the intervention period with clinical response (defined as a PDAI score reduction of ≥3) occurring in 1/10 (10%) patients in the probiotic group and 0/10 (0%) patients in the placebo group (10% * vs.* 0%, *P* = 0.32). 

In an open-label trial of 51 UC patients post ileal pouch-anal anastomosis using a fermented milk product with a blend of probiotic strains (*L. acidophilus* strain La5 + *B. lactis* strain Bb12) containing 5 × 10^10^ CFU/day [49] however, there was a reported improvement in endoscopic evaluation. In another open label trial twenty-three consecutive patients with mild pouchitis as defined using Pouchitis Disease Activity Index (scores 7–12) were treated with 3.6 × 10^12^ CFU/day of VSL#3™ for four weeks [50]. Sixteen of 23 patients (69 percent) with mild pouchitis were in remission after treatment and the median total Pouchitis Disease Activity Index scores reported before therapy improved following therapy (10 * versus* 4, *P* < 0.01). 

Thus, there is limited evidence for a role of probiotics as monotherapy for mild/moderate pouchitis at the present time. Limiting access of microbiota to the mucosa of the pouch and subsequent development of inflammation may be a key mechanism whereby probiotics provide benefit. Alternatively, changing the composition of the pouch microbiota may be important; although it is interesting that no long-term colonization of probiotic strains is achieved [51]. Thus it may not be surprising that once the deleterious microbiota have colonized within the pouch there is little a probiotic as monotherapy can do to alter the situation. A somewhat analogous situation exists for use of probiotics as monotherapy in treating *Helicobacter pylori*. The eradication rate of probiotic monotherapy was poor compared to standard triple therapy (a proton pump inhibitor + 2 antibiotics) in children colonized with *H. pylori* [52]. Interestingly, a number of studies have reported indirect evidence suggested reduced *H. pylori* colonization with probiotic monotherapy even though eradication rate is poor [53] and one study suggested reduced gastritis on biopsy [54]. The increased eradication rates of *H. pylori* using combined probiotic and antibiotic may take advantage of lower levels of pathogen in the stomach and/or decreased adverse effects of the antibiotics. Thus, if there is an analogy to be drawn for it would be interesting in future studies of patients with pouchitis requiring continuous antibiotics or very frequent use of antibiotics whether probiotics had a role following short antibiotic courses of therapy. 

#### 2.3.2. Prevention of Initial Post-Operative Onset of Pouchitis

Two trials have studied whether there is an advantage to initiate probiotics immediately following ileal pouch-anal anastomosis and both found there to be benefit to the delay in onset of development of pouchitis. One of these was a placebo-controlled trial [55] In this controlled trial at the end of one year 2 of 20 (10%) of those in the intervention arm had developed colitis as determined compared to 8 of 20 (40%, no episodes 80% * versus* 60%, *P* = 0.03) of the control arm participants using the PDAI with endoscopy. The Peto odds ratio for prevention of pouchitis by VSL#3™ compared with placebo was 4.76, 95% CI 1.16 to 19.56 [56]. 

The other randomized trial or probiotics also studied VSL#3™ in an open-label design that compared the probiotic to no treatment over a 12 month period [57]. None of the 16 patients in the group administered probiotic compared to one of 12 (8.3%, no pouchitis 100% * versus* 92%, *p* = 0.24) developed pouchitis. 

#### 2.3.3. Maintenance of Pouchitis Remission (Table 3)

The initial controlled trial for this indication was in the year 2000 using the blend probiotic product, VSL#3™ and reported on outstanding effect in prevention of the recurrence of pouchitis in patients with antibiotic-dependent pouchitis. Prior to the administration of the blend probiotic, participants in his trial were successfully treated with a combination of antibiotics (ciprofloxacin + rifaximin). At the end of the study period of 9 months, only 3 of 20 (15%) had developed pouchitis in the intervention group whereas all 20 participants in the control group had a recurrence of pouchitis and this had occurred 4 months following the antibiotics [58]. A similar result was noted in another European trial of VSL#3™ that also evaluating the prevention of recurrence of pouchitis in relapsing or chronic pouchitis patients [51]. Remission of the pouchitis was induced in these participants by administering 4 weeks of a combination of antibiotics (metronidazole + ciprofloxacin) that was followed by either VSL#3™ or a placebo. In the treatment group remission was maintained in 17 of 20 (85%) but only 1 of 16 (6%, *p* < 0.0001) on placebo. The pooled Peto odds ratio for these two studies for the combined rate of maintenance of remission with probiotic bacteria compared to placebo (97% * versus* 3%, *P* < 0.0001) was 25.39 (95% CI 10.37 to 62.17). The number needed to treat with oral probiotic therapy to prevent one additional relapse was 2 [56].

**Table 3:** Randomized trials of probiotics in prevention of onset or recurrence of pouchitis.

Participants (# Treated)	Trial Design	Probiotic (Strains)	Dosing (CFU/day)	Trial Length (Months)	References
40 (20)	DBRPC	Blend (VSL#3™)	9 × 10^11^	12	[55]
31 (16)	R	Blend (VSL#3™)	9 × 10^11^	12	[57]
40 (20)	DBRPC	Blend (VSL#3™)	1.8 × 10^12^	9	[58]
36 (20)	DBRPC	Blend (VSL#3™)	9 × 10^11^	12	[51]

Same footnotes as Table 1.

In contrast, an open label trial by Shen and colleagues [59] reported lesser responses. In their trial, 31 subjects were prescribed a 2-week treatment of a single antibiotic (ciprofloxacin) followed by VSL#3™. Also in contrast to the other studies, the VSL#3™ was bought by patients rather than be supplied through the study. Probiotic therapy was stopped by 9 of 31 (29%) seven weeks into therapy and 25 of 31 (81%) by 8 months had discontinued the probiotic because of failure to prevent pouchitis (*n* = 23) or side effects of the probiotic administration (*n* = 2). Only 6 of 31 (19%) did not develop clinical evidence of pouchitis by the end of the 8-month trial period. Even among these 6 subjects endoscopy revealed some level of pouch inflammation. In this trial [59], there was a single antibiotic administered and endoscopy was not performed prior to probiotic administration to ensure pouch inflammation had completely resolved. 

A recent clinical practice guideline on management of pouchitis [46] has suggested that for those patients with prompt recurrence of pouchitis following antibiotic usage or having multiple recurrences of pouchitis despite antibiotics either VSL#3™ or chronic use of antibiotics but does not suggest probiotics for acute treatment of pouchitis. 

## 4. Crohn’s Disease

### 4.1. Treatment of Active Crohn’s Disease Inflammation

There is a paucity of studies that have investigated use of probiotics to settle active inflammation. For two open label studies [60,61], probiotics (using combination of *B. breve* + *L. casei* + *B. longum* + prebiotics or *L. rhamnosus* GG, respectively) were added to immunomodulators and corticosteroids. In the former study [60] in 7 of 10 patients were reported to respond as determined by Crohn’s Disease Activity Index scores with most noticeable improvement in the diarrhea. However, there was no improvement in inflammation as measured by erythrocyte sedimentation rate (ESR) and C-reactive protein (CRP). In the open label trial using *L. rhamnosus* GG [61], 3 of the 4 children were reported to have improved Pediatric Crohn’s Disease Index (PCDAI) scores or serial determinations over the 6 months of the trial. Specifics with regards to ESR or CRP are not reported but the ESR is a component of the PCDAI [62]. 

A placebo-controlled trial using *L. rhamnosus* GG was the sole study included in a Cochrane review of efficacy of probiotic supplementation for the induction of remission in CD that met the inclusion criteria of being a randomized controlled trials of participants with Crohn’s disease whose disease was active at the time of entry into the study [63]. There were a total of 11 participants in the study and subjects received antibiotics and concurrent therapy of corticosteroids and some methodological concerns were raised. Four of 5 patients in the probiotic group achieved remission compared to 5 of 6 in the placebo group (OR 0.80; 95% CI 0.04 to 17.20). Thus, this one small study did not show that probiotics had any effect in treating active Crohn’s disease. At best, one could say there is insufficient evidence to make any conclusions about the effectiveness of probiotics for treatment of active Crohn’s disease.

### 4.2. Probiotics as Maintenance Therapy for Crohn’s Disease

Initial randomized trials (See Table 4) in Crohn’s disease were reported with probiotics used as sole maintenance therapy following corticosteroid therapy [64] or in combination with lower does of 5-aminosalicylate therapy compared to controls for maintenance therapy in those already in remission [65]. Subsequent trials have focused on *L. rhamnosus* strain GG for maintenance therapy following induction of remission with corticosteroids [66] and maintenance of remission with probiotic used as additional maintenance therapy [67]. There were no differences in the number of relapses in patients receiving *E. coli* Nissle compared to the placebo (*P* = 0.11), *Saccharomyces boulardii* (1 g/day) plus mesalazine (2 g/day) compared to mesalazine alone (3 g/day) (*P* = 0.08), or patients with patients with remission induced medically receiving *L. rhamnosus* GG (*P* = 0.77).

**Table 4:** Randomized trials of probiotics in Crohn’s disease maintenance.

Participants (# Treated)	Trial Design	Probiotic (Strains)	Dosing (CFU/day)	Trial Length (Months)	References
28 (16)	DBRPC	Single strain (*E. coli* Nissle 1917)	5 × 10^10^	12	[64]
32 (16)	R	Single strain (*S. boulardii*)	N/A	6	[65]
11 (5)	DBRPC	Single strain (*L. rhamnosus* strain GG)	2 × 10^9^	6	[66]
75 (39)	DBRPC	Single strain (*L. rhamnosus* strain GG)	2 × 10^10^	24	[67]

Same footnotes as Table 1.

**Figure 1:**
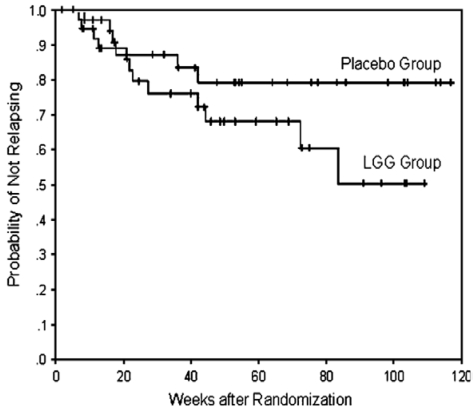
Kaplan-Meier survival curve showing the probability of staying relapse-free during the duration of the study treatment duration for participants administered *L. rhamnosus* GG or placebo. Reproduced with permission from John Wiley and Sons [67].

In the largest maintenance trial to date, Bousvaros *et al.* [67] also reported no difference in the proportion of those developing relapse on *L. rhamnosus* strain GG 2 × 10^10^ CFU/day (31%; 12 of 39) or placebo (17%; 6 of 36, *p* = 0.18). The time to relapse is shown in Figure 1, and although the probiotic group trended to a shorter time to relapse, comparison between it and the control group on placebo was not statistically different (*p* = 0.10).

Two other studies reported in abstract are reviewed in Cochrane review on probiotics for Crohn’s maintenance. In the review no difference in subjects receiving *L. rhamnosus* GG plus mesalazine compared to those receiving the same level of maintenance therapy without probiotic as determined by CDAI in one abstract and no difference in endoscopic relapse using for VSL#3™ compared to mesalamine alone in the other abstract [68]. This must be taken in the context that mesalamine has no benefit for maintenance of Crohn’s disease in itself [69]. 

### 4.3. Probiotics for Prevention of Post-Operative Crohn’s Disease Recurrence

Another aspect of Crohn’s disease that has been studied is that of prevention of recurrence of disease following surgical resections and details as to the trials are included in Table 5. There are however, 5 randomized trials [70,71,72,73,74] that have been published. Three of the trials involved single probiotic strains being administered and 2 trials included combination probiotics. In the *L. rhamnosus* GG trial [70], 9 of 15 (60%) in *L. rhamnosus* GG group in clinical remission had endoscopic recurrence and 6 of 17 (35%) in placebo group in clinical remission had endoscopic recurrence (*p* = 0.297). In the first *L. johnsonii* LA1 trials [71], at 6 months endoscopic recurrence was seen in 21 of 43 (43%) in *L. johnsonii* LA1 group and 30 of 47 (64%) of placebo group and (*p* = 0.15). The second *L. johnsonii* LA1 trial [72] also showed similar overall endoscopic scores between the probiotic and placebo groups (*P* = 0.48) and similar numbers of those with severe endoscopic recurrence with 21% of those taking *L. johnsonii* LA1 *versus* 15% taking placebo (*P* = 0.33). None of the secondary outcomes (clinical recurrence, histological score, C-reactive protein) showed any difference either. Similarly, the 2 studies using a blend of probiotics failed to reveal any differences between treatment and placebo groups for endoscopic recurrence [73,74]. A meta-analysis [75] of the effects of probiotics as a class suggested that their effect was no different than placebo. The relative risk of clinical recurrence with any probiotic relative to placebo (*n* = 213) was 1.41 (95% CI 0.59 to 3.36), any endoscopic recurrence (*n* = 333) was 0.98 (95% CI 0.74 to 1.29) and severe endoscopic recurrence (*n* = 213) was 0.96 (95% CI 0.58 to 1.59). 

**Table 5:** Double-blind placebo controlled trials of probiotics for prevention of post-operative Crohn’s disease recurrence.

Participants(# Treated)	Probiotic (Strains)	Dosing (CFU/day)	Trial Length (Months)	References
45 (23)	Single Strain (*L. rhamnosus* GG)	1.2 × 10^9^	12	[70]
98 (48)	Single Strain (*L. johnsonii* LA1)	4 × 10^9^	6	[71]
70 (34)	Single Strain (*L. johnsonii* strain LA1)	10^10^	3	[72]
30 (2)	Blend Probiotics + Prebiotics (*P. pentoseceus*, *L. raffinolactis*, *L. paracasei* susp paracasei 19 and *L. plantarum* 2362)	10^10^	24	[73]
120 (58)	Blend Probiotics (VSL#3™)	1.8 × 10^12^	3	[74]

Same footnotes as Table 1.

## 5. Associated Conditions

### 5.1. Arthralgia

In an open label trial with a dropout rate of 45%, 16 patients with either Crohn’s disease or ulcerative colitis completed a 3-month trial of ingesting 9 × 10^11^ CFU/day of a blend probiotic (VSL#3™) to assess whether there was a clinical improvement in arthralgia [76]. Participants had quiescent IBD at entry and no clinical or laboratory evidence of arthritis, were not taking non-steroidal anti-inflammatory medications and other medications were unchanged. An improvement in peripheral but not axial arthralgia was reported using an articular index score but no joint pain improvement as reported using a patient-completed visual analog scale.

### 5.2. Spondylarthropathy

In an interesting internet-based randomized control trial [77] of probiotic in patients with spondylarthropathy included some 7% with concomitant IBD. The primary aim of the study was to determine whether an internet-based trial of a complementary and alternative medicine could fulfill the revised CONSORT (Consolidated Standards of Reporting Trials) statement quality checklist for reporting of RCTs. However a secondary aim was to study the effect of probiotics on improving well-being. Well-being was measured by self-assessment using a visual analog scale and 96 of 147 (65%) of people randomized to receive a blend probiotic completed a 3-month trial. No statistically or clinically significant difference between placebo and probiotic groups in terms of global well-being was determined in the study [77].

### 5.3. Sclerosing Cholangitis

Fourteen participants with concurrent IBD were randomized to treatment with a blend probiotic (*L. acidophilus*, *L. casei*, *L. salivarius*, *L. lactis*, *B. bifidum and B. lactis*; total daily dose of 10^10^ CFU/day) or placebo during 3 months in a double-blind crossover design that included a 1-month washout period [78]. The subjects remained on their ursodeoxycholic acid. The results of this study showed no evidence of a beneficial effect of the probiotics on PSC-related symptoms, serum liver biochemistry or liver function.

## 6. Summary

It is important to not generalize reports of positive benefit from specific strain studies to species effects as it is equally as important not to generalize negative reports of a specific strain to a species effect. In addition, all reviews clearly acknowledge the need for further studies with regards to dosing, duration of therapy, delivery methods and whether blends of different strains of probiotics offer any benefit over single strains. That being said, clinical practice guidelines are now including a probiotic (*i.e.*, VSL#3™) for recurrent and relapsing antibiotic sensitive pouchitis. Use of probiotics in UC is provocative and suggests potential for benefit in select patients but concerns remain about proof from trials. Costs can be a barrier since few funders of health care (e.g., North American insurance companies or Government plans) cover the costs of probiotics for maintenance therapy. Certainly for those intolerant to 5-aminosalicylate products specific probiotic products would appear to have more potential for modest effect in maintenance of remission for those with mild/moderate disease activity. 

In contrast, there is no evidence that patients with Crohn’s disease will benefit from ingestion of probiotics for any aspect of their disease whether it is for treating active disease, maintaining remission or preventing post-operative recurrence of disease. Neither is there benefit reported in some of the IBD associated conditions in the small trials that have been reported to date. 

One could speculate that since CD involves inflammation of the full thickness of the mucosa, therapy such as microbes interacting at the mucosal-luminal interface would be less likely to have efficacy than with ulcerative colitis. However, this may be simplistic as more likely modulating the bacteria luminal mucosal epithelial cell interface is not as important in Crohn’s disease as innate immune defects that don’t exist in ulcerative colitis. Despite these dismal results for CD, there are a number of novel strategies and approaches to consider that may hold hope for this form of therapy. Currently, most probiotics include various strains of *Lactobacillus* or *Bifidobacterium*. Perhaps microorganisms that have been found to be deficient in microbiota studies of CD patients such as *Faecalibacterium prausnitzii* [79] or others found to be increased with certain diets and have an anti-inflammatory effect such as *Enterococcus durans* [80,81] will prove to provide a CD health benefit. In addition, genetics is an integral part of the pathogenesis of CD and genetic make-up of the human host has not been explored in probiotic trials. Perhaps combining specific microbes with a specific host genotype might be necessary for efficacy. Another possibility may be the use of genetically altered microorganisms to deliver specific genes to the mucosa microbiome might also provide a health benefit [82,83], albeit administration of genetically modified microbes to humans will require significant safety studies. 

Given the current situation of parental and self-prescribing of alternative care products including those described as probiotics, it behooves those providing care for IBD patients to know exactly all prescription and non-prescription items being administered to IBD patients so when problems in care are occurring comprehensive strategization of this care can be co-coordinated. Among the most serious clinical scenarios to consider is that in which a patient is initiating immunosuppressive therapy. While most patients undergo the initiation of immune altering medications without incident, immunosuppression is a possibility. There is no evidence for the use of probiotics in any form of severe IBD and little clinical experience in the use of probiotics administration to severely immunocompromised IBD patients. However, in ill patients in ICU settings fungemia has developed from the use of *S. boulardii* as probiotic [84] and sepsis from a *Lactobacillus* strain has also been reported in an ulcerative colitis patient [85]. With commercialization of probiotics ahead of scientific and clinical investigation, as practitioners we should demand that the various aspects of IBD care are critically appraised before encouraging patients to ingest undocumented probiotic products as therapy in IBD.
